# Comparing image analysis approaches versus expert readers: the relation of knee radiograph features to knee pain

**DOI:** 10.1136/annrheumdis-2018-213492

**Published:** 2018-08-01

**Authors:** Luca Minciullo, Matthew J Parkes, David T Felson, Timothy F Cootes

**Affiliations:** 1 Division of Informatics, Imaging & Data Sciences, The University of Manchester, Manchester, UK; 2 NIHR Manchester Musculoskeletal Biomedical Research Centre, Manchester University Hospitals NHS Foundation Trust, Manchester Academic Health Science Centre, Manchester, UK; 3 Clinical Epidemiology Research and Training Unit, Boston University School of Medicine, Boston, Massachusetts, USA

**Keywords:** knee osteoarthritis, osteoarthritis, arthritis

## Abstract

**Objectives:**

The relationship between radiographic evidence of osteoarthritis and knee pain has been weak. This may be because features that best discriminate knees with pain have not been included in analyses. We tested the correlation between knee pain and radiographic features taking into account both image analysis features and manual scores.

**Methods:**

Using data of the Multicentre Osteoarthritis Study, we tested in a cross-sectional design how well X-ray features discriminated those with frequent knee pain (one question at one time) or consistent frequent knee pain (three questions at three times during the 2 weeks prior to imaging) from those without it. We trained random forest models on features from two radiographic views for classification.

**Results:**

X-rays were better at classifying those with pain using three questions compared with one. When we used all manual radiographic features, the area under the curve (AUC) was 73.9%. Using the best model from automated image analyses or a combination of these and manual grades, no improvement over manual grading was found.

**Conclusions:**

X-ray changes of OA are more strongly associated with repeated reports of knee pain than pain reported once. In addition, a fully automated system that assessed features not scored on X-ray performed no better than manual grading of features.

## Introduction

Despite considerable effort, the existence of pain has not found to be strongly correlated with radiographic osteoarthritis (OA).^1–5^ First, this poor agreement may be because the global measures of radiographic disease that are used in these studies are insensitive to specific features that are better correlated with pain than global scores. Second, these studies have generally been limited to uniplanar radiographs and therefore miss features that are correlated with the presence of pain. Third, some individuals may have knee pain as part of a syndrome of widespread pain and do not have OA. Last, knee pain is often transient and radiographic disease may be more likely in persons in whom it is consistently reported.

Previous studies involve the investigation of correlation between individual structural features such as osteophytes and joint space narrowing (JSN)^2 5 6^ and pain. Felson and colleagues^7^ gave an alternative definition of OA based on a combination of structural features and showed a modestly improved correlation with pain. Minciullo *et al* 8 used constrained local models (CLMs) to find landmark points for the knee joint in both lateral and PA radiographs and extracted features related to bone shape, texture and their combination to predict onset of knee pain, showing a weak correlation with structural features and suggesting that the lateral view contains features that are significantly more discriminative compared with the PA view. Galvan-Tejada *et al* 1 used radiographs from the osteoarthritis initiative (OAI) to prove that osteophytes are early predictors of joint pain, while joint space reduction is not clearly associated with future joint pain.

The objective of our work was to determine the correlation between knee pain and various sets of radiographic features of OA obtained at the time of the pain report, using both features automatically extracted from knee radiographs and manual grades assigned by clinicians. To do so, we built random forest classifiers using a large collection of features and, unlike most previous works, we used both posteroanterior and lateral radiographs. We also tried combining structural features with image independent features such as age and Body Mass Index (BMI), which are known to increase risk of developing OA.^4^ Furthermore, we tried to exclude from the study people who were experiencing widespread pain, under the assumption that such pain may not be due to OA.

## Methods

Images were taken from the Multicentre Osteoarthritis Study (MOST) dataset.^9^ Bilateral PA standing flexed and unilateral weight bearing, flexed lateral radiographs were obtained at baseline. At baseline, subjects were asked three times whether they had knee pain, aching or stiffness on most of the last 30 days. First, a telephone screening (TScreen) done roughly 2 weeks before the clinic visit was performed to check eligibility criteria. Second, before the visit, participants filled a Self-Assessed Questionnaire (SAQ) at home. Last, an interview was done as part of the clinical visit (Clinic). We used the TScreen, the Clinic and SAQ variable together to create a measure we called ‘Consistent Pain’. By consistent pain, we meant selecting participants who gave the same binary score at all three time points. We used data on right knee pain and right knee imaging findings. We characterised widespread pain as present when the person reported frequent pain above and below the waist and on both sides of the body and in the axial region. In our experiments, we only considered data from the baseline.

The radiographic grades used in our work were assigned by central readers as part of the MOST study protocol. Two types of features were used in our experiments.

- Manual grades for features of OA assigned by readers during the MOST study. We used scores for all the features that were read on both the PA and lateral views, including Kellgren-Lawrence (KL grades.
- Shape, texture and appearance features automatically extracted using CLMs to find landmark points in radiographs.

**Figure 1:**
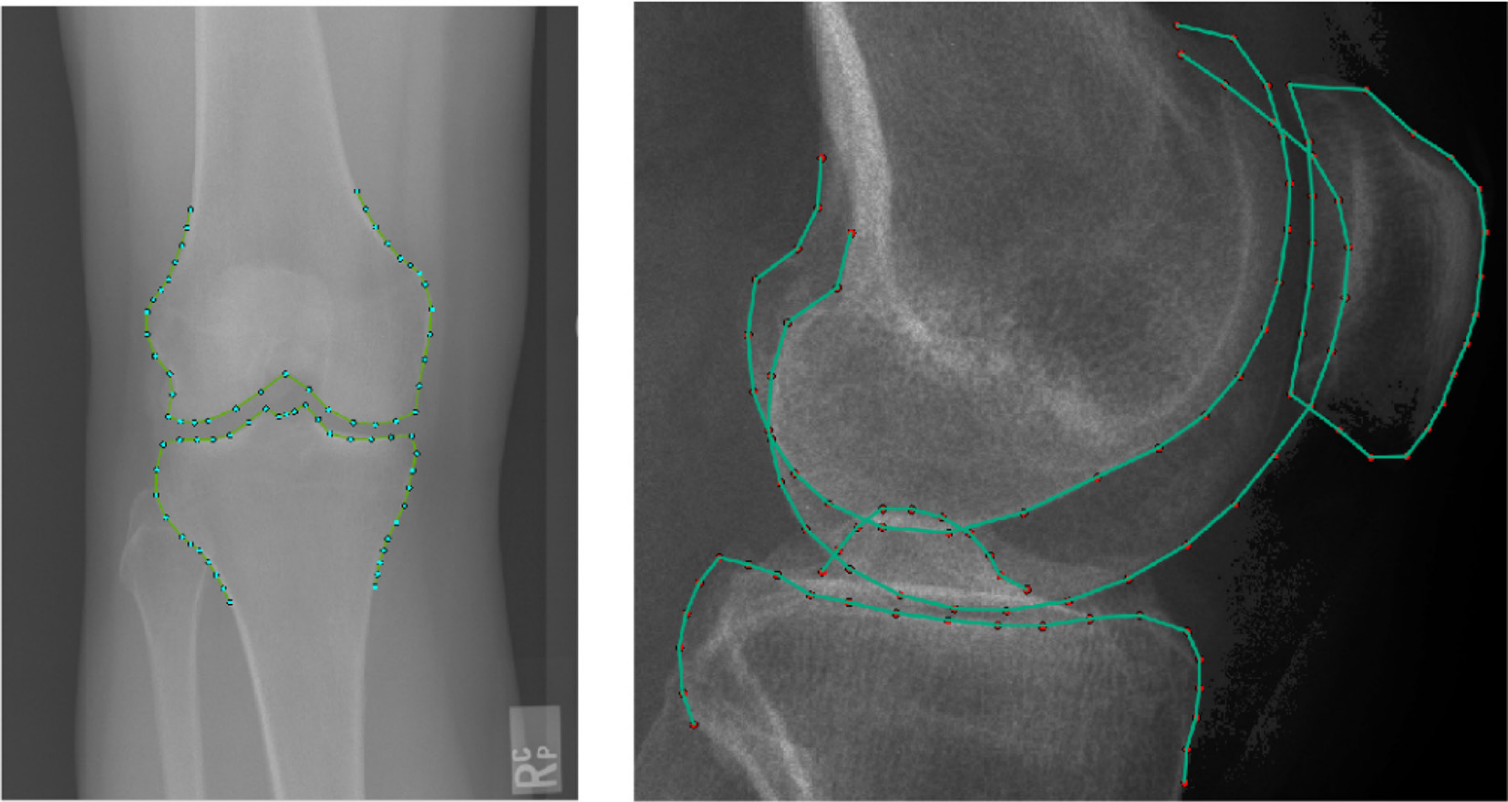
Our model for lateral radiographs (right) was made of four subshapes: the patella (21 points), the lateral femoral condyle (24 points), the medial femoral condyle (25 points) and the tibia (32 points). We considered the femur as the union of the two femoral condyles (49 points). The PA model (left) was made of two shapes: the femur and the tibia (37 points each, a total of 74 points).

CLMs have been successfully applied in medical imaging on a large variety of radiographic images.^8 10 11^ Our models for lateral and PA radiographs are shown in figure 1.

### Appearance model

We extracted features by building an appearance model. Combined appearance models 12 are an attempt to better use textural information and are based on a statistical model that uses shape as one of its components. Such a model incorporates non-redundant information of the shape and the texture of the object of interest. For full details see Minciullo *et al*.^8^

### Object detection and shape model matching

We developed an automatic system to locate the outlines of the bones in both radiographic views. It first finds the position of a bounding box around the joint and then refines this with a shape model matching algorithm—for full details, see Minciullo *et al*, Gall *et al* and Lindner *et al*.^11 13 14^

### Analysis approach

First, we tested the relation of individual features and KL grades with the presence of pain. All the experiments were performed training and testing a random forest classifier with 40 trees, running a 5-fold cross validation with five repeats. We used the area under this receiver operator characteristic (ROC)curve to determine the relation of knee pain with radiographic features. We report the SD of the performance evaluated using the AUC over five repetitions.

We compared a single question for frequent knee pain with the same question administered three times in relation to the baseline MOST visit. For the latter approach, we compared people who consistently reported knee pain to those who did not report knee pain at any of the three time points.

The subsequent analyses tested whether automated image analysis generated a higher AUC than did a combination of manually scored features. In addition, we tested whether a combination of information provided by image analysis and manual grading improved on the ROC curve area compared with manual grading alone.

χ² tests were used to assess the difference in AUC between the manual scoring (as the gold standard), adding BMI and sex and the best fully automated model. The p value of 0.05 or below was selected to indicate that the ROC curve differed from the gold standard statistically significantly.

## Results

We studied 2756 MOST participants at baseline. The mean age was 62.3 years (SD 8) and mean BMI was 30.7 (SD 5.9). Of those studied, 60% were women.

### Testing individual radiographic features

There are 36 individual radiographic features (mostly Osteoarthritis Research Society International (OARSI) grades) scored from the PA and lateral radiographs (listed in table 1). For each, we measured the AUC when using the grade as the only feature in a classifier. We observe that KL grade, osteophytes, JSN and sclerosis were the most discriminative with the KL grade achieving the best result. On the other hand, chondrocalcinosis, cyst, attrition and ossification of the patella-tendon performed no better than chance. While some of these results were expected, bone attrition (as MRI feature) was previously found to be associated with OA pain.^5^

**Table 1:** Testing each radiographic feature individually using the pain score reported during the visit (Clinic)

Variable	AUC (%)
Chondrocalcinosis (OARSI grades 0–1) PF joint on LA view	50±0.3
Osteophytes (OARSI grades 0–3) femur anterior PF joint on LA view	58.3±0.2
Osteophytes (OARSI grades 0–3) femur posterior PF joint on LA view	60±0.5
Joint space narrowing (OARSI grades 0–3) lateral TF compartment on LA view	55.2±0.2
Joint space narrowing (OARSI grades 0–3) medial TF compartment on LAT view	59.1±0.3
Effusion (OARSI grades 0–1) PF joint on LA view	56±0.3
Kellgren & Lawrence (grades 0–4) on PA view	**64.8±0.1**
Chondrocalcinosis (OARSI grades 0–1) lateral TF compartment on PA view	50.4±0.3
Cyst (OARSI grades 0–3) femur lateral TF compartment on PA view	50.6±0.3
Osteophytes (OARSI grades 0–3) femur lateral TF compartment on PA view	60.2±0.2
Sclerosis (OARSI grades 0–3) femur lateral TF compartment on PA view	54.3±0.3
Joint space narrowing (OARSI grades 0–3) lateral TF compartment on PA view	54.9±0.3
Attrition (OARSI grades 0–1) lateral TF compartment on PA view	50.6±0.2
Cyst (OARSI grades 0–3) tibia lateral TF compartment on PA view	50.6±0.2
Osteophytes (OARSI grades 0–3) tibia lateral TF compartment on PA view	60±0.2
Sclerosis (OARSI grades 0–3) tibia lateral TF compartment on PA view	54.2±0.3
Chondrocalcinosis (OARSI grades 0–1) medial TF compartment on PA view	50.7±0.1
Cyst (OARSI grades 0–3) femur medial TF compartment on PA view	50.8±0.3
Osteophytes (OARSI grades 0–3) femur medial TF compartment on PA view	61±0.3
Sclerosis (OARSI grades 0–3) femur medial TF compartment on PA view	57.7±0.2
Joint space narrowing (OARSI grades 0–3) medial TF compartment on PA view	57.7±0.2
Attrition (OARSI grades 0–1) medial TF compartment on PA view	52.1±0.2
Cyst (OARSI grades 0–3) tibia medial TF compartment on PA view	51.5±0.3
Osteophytes (OARSI grades 0–3) tibia medial TF compartment on PA view	59.7±0.2
Sclerosis (OARSI grades 0–3) tibia medial TF compartment on PA view	58.3±0.4
Ossification (OARSI grades 0–3) patella tendon lower PF joint on LA view	49.5±0.1
Ossification (OARSI grades 0–3) patella tendon upper PF joint on LA view	50±0.6
Ossified loose body (OARSI grades 0–1) femur posterior PF joint on LA view	52.2±0.3
Ossification of quadriceps femoris insertion (OARSI grades 0–3) PF joint on LA view	51±0.3
Cyst (OARSI grades 0–3) PF joint on LA view	51±0.2
Joint space narrowing (OARSI grades 0–3) PF joint on LA view	53.2±0.3
Sclerosis (OARSI grades 0–3) PF joint on LA view	53.1±0.4
Osteophytes (OARSI grades 0–3) patella inferior PF joint on LA view	59.5±0.2
Osteophytes (OARSI grades 0–3) patella superior PF joint on LA view	60.1±0.3
Osteophytes (OARSI grades 0–3) tibia anterior PF joint on LA view	55.5±0.1
Osteophytes (OARSI grades 0–3) tibia posterior PF joint on LA view	59.4±0.3

Bold values correspond to the best results.

### Testing combinations of radiographic features

Next we combined all the available manually graded features considering all pain scores defined previously (see table 2). Removing participants with widespread pain made little difference to the manual model, while adding BMI and sex significantly improved the ROC for both Clinic and SAQ pain. The AUCs for consistent pain were higher, especially for manual grades (eg, 73.9 vs 62.8–66.7) and the SD around these estimates were narrow.

**Table 2:** Performance of R classifiers when using all the available clinician grades as features

Features	# Samples	AUC±SD	P value vs Referent
Telephonic screening interview			
Manual grades	2756	62.8±0.4	Referent
Manual Grades+Gender+BMI		66±0.5	**<0.001**
Best automated		63.8±0.2	0.15
Manual+Automated		65.6±0.3	**<0.001**
Removing widespread pain	1374	61±0.2	0.51
Clinic			
Manual grades	2756	66.4±0.2	Referent
Manual Grades+Gender+BMI		68.8±0.2	**<0.001**
Best automated		65.6±0.9	0.29
Manual+Automated		63±0.3	**0.01**
Removing widespread pain	1374	61±0.2	**0.02**
Self-assessed questionnaire (HOME)			
Manual grades	2756	66.7±0.3	Referent
Manual Grades+Gender+BMI		68.9±0.4	**<0.001**
Best automated		67.7±0.3	0.30
Manual+Automated		68±0.2	**0.05**
Removing widespread pain	1374	69±0.2	0.10
Consistent pain (answered yes to pain at all time points)			
Manual grades	1066	73.9±0.5	Referent
Manual Grades+Gender+BMI		**76.1±0.2**	**0.01**
Best automated		73.1±0.7	0.97
Manual+Automated		75.6±0.6	0.14
Removing widespread pain	565	78±1	**0.04**

The p values compare the AUCs with the referent in that pain group. For example, for telephone screening, compared with manual grades, none of the other approaches was significant.* Sometimes these p values show significantly worse AUCs than the referent.

*Comparison eliminating participants with widespread pain was performed using manual grades+Gender+BMI features.

Bold values correspond to the best results.

When working with each pain score individually, the results show that using the best performing automated model gives results that were not significantly different from those of manual grades.

The combination between manual grades and the appearance features extracted from the model was not more discriminative than manual grades alone.

## Discussion

We found that identifying persons with consistent knee pain using manually read radiographic features gave the highest AUC. The best model using features computed automatically from the images could be used to discriminate pain from non-pain, without significant loss in AUC compared with using manual grades. Furthermore, removing participants with widespread pain made the classification better for consistent pain.

The main strengths of this work are (1) the size of the dataset used, one order of magnitude larger than most similar studies, (2) we presented the most comprehensive corpus of experiments looking at correlations between radiographs and symptomatic OA, using both PA and lateral view images, therefore including PF joint^15^ and posterior compartments and (3) we explored for the first time OARSI grades of the lateral view of the MOST study and their combination with other radiographic features.

Limitations are the absence of skyline view radiographs, which could provide further discriminative information, but were not acquired during the MOST study. We did not have information on the duration of knee pain and examined only cross-sectional, not prospective, data. The correlations with more chronic or persistent pain and the impact of other risk factors remain to be determined. The extension of this work to MRI features, that have been shown to be more correlated with symptoms, is a promising addition for future work. Another area of interest will be the search for patterns in fMRI related to pain perception in participants with OA or at risk of developing it.
